# Burden and Help-Seeking Behaviors Linked to Problem Gambling and Gaming: Observational Quantitative and Qualitative Analysis

**DOI:** 10.2196/26521

**Published:** 2021-11-26

**Authors:** Amandine Luquiens, Cora von Hammerstein, Amine Benyamina, Pascal Perney

**Affiliations:** 1 Universitary Hospital of Nîmes University of Montpellier Nîmes France; 2 UVSQ, CESP, INSERM Paris-Saclay University University of Paris-Sud Villejuif France; 3 Addictions Department Paul Brousse Hospital, APHP Paris-Saclay University Villejuif France; 4 University Research Team EA4360 APEMAC (Health Adjustment, Measurement and Assessment, Interdisciplinary Approaches) University of Lorraine Metz France

**Keywords:** gambling, gaming, helpline, burden, relatives, qualitative research

## Abstract

**Background:**

Models based on the uniqueness of addiction processes between behavioral addictions are highly contentious, and the inclusion of gaming disorder in the addiction nosography remains controversial. An exploratory approach could clarify a hypothesized common and subjectively identifiable process in addictive behaviors and the necessarily different expressions of the disorder due to behavior specificities, in particular the sociocultural characteristics and profiles of users.

**Objective:**

The aim of this study was to describe the nature of contacts to a help service by exploring commonality and specificities of burden and help-seeking for problem gambling or gaming.

**Methods:**

This was an observational quantitative-qualitative study. We included all contacts (ie, online questions and contacts by phone or chat when the helper completed a summary) to a helpline for gamers, gamblers, and relatives over a 7-year period. We constituted a text corpus with online questions and summaries of contacts by phone or chat. We collected basic sociodemographic data, including the device used to contact the service (phone or internet), contacting the service for oneself (“user”) or being a relative of a user and type of relative, gambling (yes/no), gaming (yes/no), and age and sex of the gambler/gamer. We describe the corpus descriptively and report the computerized qualitative analysis of online questions, chat, and summary of phone calls. We performed a descendant hierarchical analysis on the data.

**Results:**

A total of 14,564 contacts were made to the helpline, including 10,017 users and 4547 relatives. The corpus was composed of six classes: (1) gaming specificities, (2) shared psychological distress and negative emotions, (3) the procedure for being banned from gambling, (4) the provided help, (5) gambling specificities, and (6) financial problems.

**Conclusions:**

Negative emotions and shared distress linked to gambling and gaming support current scientific consensus that these behaviors can produce psychological distress in se; however, meaningful differences were observed in core symptoms of addiction between gamers and gamblers, beyond specificities related to the behavior itself: loss of control was elicited in the class corresponding to gambling specificities and not by gamers and their relatives.

## Introduction

The International Classification of Diseases (ICD)-11 recognizes two behavioral addictions [1]: gaming and gambling disorders. The clinical descriptions of these disorders comprise three main dimensions: impaired control, increasing priority given to gambling/gaming, and continuation despite the occurrence of negative consequence. This classification implies common processes between different types of addiction in the development and maintenance of addiction. This commonality is proposed by models of behavioral addictions, such as the Interaction of Person-Affect-Cognition-Execution model [2], which presents addictive behaviors as consequences of the interactions between individual vulnerability factors, affective and cognitive responses to specific behavior, and executive functions. This model includes specificities related to the behavior itself that render them more attractive to individuals with certain psychological profiles. However, it does not include the social dimension of behaviors accounting for a social group’s greater propensity to use the same behavior as a commodity associated with the group’s identity, in contrast with other sociological approaches [3]. Models based on the uniqueness of addiction processes between behavioral addictions are highly contentious, and the inclusion of gaming disorder in the addiction nosography remains controversial [4]. Diagnosis criteria are debated [5], and some authors still consider problem gaming as a symptom of another mental disorder [6].

Beyond these models based on the uniqueness of addictive disorders, comparisons between addictive behaviors in the same study are scarce. Furthermore, the literature lacks a clear picture of the differences between potential addictive behaviors, particularly in the representation of the behavior itself, the associated disorder, and the inner experience and perception of the disorder by users and their relatives. Few studies have made comparisons between gaming and gambling. A previous qualitative study found significant overlap in the experience of people living with a substance use problem and a behavioral addiction/problem [7]. However, gaming and gambling differ at various levels: at the clinical level, assessing the importance of depression as a risk factor in gaming [8]; at the neuropsychological level, in terms of delay discounting and decision making [9]; at the neural level; and in the pharmacodynamic response to certain treatments [10]. However, there are also specificities linked to different profiles of users between the behaviors as commodities. Gaming is more widespread in adolescents and young adults, and the age of onset is earlier than that in gambling, where the average age of onset is around 34 years and the practice is prohibited among minors in most countries [11]. For instance, in a study among 824 adolescents, a prevalence of 3% was obtained of at-risk gaming, with a mean age of 14.5 years, and the majority reported a symptom course greater than 12 months [12]. This younger age range leads to specific cognitions in problem gamers [12].

These differences in profiles support the biopsychosocial model in addictions [13], where addictive behavior as a commodity is consumed by different populations and related disorders have a different representation in the general population [14] with different degrees of stigma, leading to specific clinical pictures and specific complaints from the addict population and relatives. However, these elements are surprisingly absent from the classifications, as illustrated by the absence of age specification in the clinical description of the ICD-11 for gaming disorder [15]. It is therefore important to focus on the difficulties experienced and described by the users themselves and their relatives [16]. The importance of exploratory approaches and qualitative research has also been emphasized by several authors [17,18].

The treatment gap is considerable in behavioral addictions, with fewer than 12% of pathological gamblers seeking help from a health professional [19]. The epidemiological data on gaming disorder are weak, but the prevalence is estimated to range from 0.5% to 10%. The care network and pathways are still being structured. No quantified data exist on the treatment gap in gaming disorder, but it is assumed to be even wider [20]. Nonface-to-face, telephone, and online support devices could help reduce this treatment gap [21], and could further help to collect information to describe a population to which clinicians and researchers have no other access. The debate over the criteria for gaming disorder could be redressed by the description of gaming difficulties by self-diagnosed problem gamers seeking help. There are also minimal data on nonface-to-face help-seekers in gambling and particularly in gaming. The current knowledge on gambling shows the preference of young gamblers for online devices [22], along with the greater reluctance among women to request help and their more difficult access to treatment [22-24]. A study of 168 gamers voluntarily contacting a help service [25] demonstrated the association of mood symptoms and severity of gaming disorder. However, no study has yet explored gaming- and gambling-related burdens collected through the same approach. An exploratory approach could clarify a hypothesized common and subjectively identifiable process in addictive behaviors and the necessarily different expressions of the disorder due to behavior specificities, in particular the sociocultural characteristics and profile of users (eg, age, sex).

We performed a quantitative-qualitative analysis of all contacts to a nonface-to-face help service over 7 years. All contactors voluntarily called or wrote to the help service asking for help or counseling regarding gaming or gambling. No diagnostic or screening assessment was performed or required to access the help service. Our aim was to explore, without a priori assumptions, the burden of gaming and gambling cited by contacts to a help service by phone and internet. Our hypothesis was that we would find both commonalities and specificities in the burden linked to gaming and gambling problems from the perspectives of users and their relatives.

## Methods

### Population and Data Collected

We report the computerized qualitative analysis of all contacts to a public national help service in France for gamers, gamblers, and their relatives since its creation in 2010 until 2018. This governmental help service, “Joueurs Info Service,” is part of a global plan for information and prevention on alcohol, substance, gaming, and gambling addictive behaviors. This is a national remote help service for gambling, gaming, and other addictions, and is also in charge of listing, updating, and making available to the public the national directory of specialized addiction structures. “Joueurs Info Service” is based on the rules of anonymity, confidentiality, neutrality, and nonjudgment in its missions of information, advice, support, and guidance of the public. Helpers are trained psychologists specialized in addictive behaviors. They empathically and actively listen to contactors and can refer them to another health facility if appropriate following their own clinical judgment. Summaries are written for phone calls and chats with helpers. These summaries do not follow a particular plan, and the level of detail and content of the summary is left to the discretion of the helpers, although they are encouraged to document the contact as exhaustively as possible. A single number and website for “Jeu Info Service” (in French, “jeu” encompasses both gambling and gaming) allows gamers, gamblers, and their relatives to talk to professional helpers by phone, chat, or written question with delayed answers. The same helpers respond to gambling and gaming contacts. We included all contacts from users and their relatives to the help service by phone when the helper completed a summary and online (ie, chat when the helper completed a summary and questions) when the helper considered contacts to be in the scope of the help service (ie, a problem with gaming and gambling: n=17,440 of a total 97,350 contacts). Nonincluded contacts were categorized by helpers as not within the scope of the helpline and were most commonly prank calls and errors (gamblers looking for the technical hotline of gambling websites). Included contacts were then people reporting having a problem with gambling, gaming, or a relative’s gaming or gambling behavior, and seeking help or counseling.

The merged corpus was composed of summaries of calls and chat discussions written by helpers, and the content of questions written directly by contactors. In the manuscript, the word “user” will designate both gamblers and gamers. Basic sociodemographic data were collected, including the device used to contact the service (phone or internet), contacting the service for oneself (“user”) or being a relative of a user and the type of relative, gambling (yes/no), gaming (yes/no), age of the gambler/gamer, age category (<25, 25 to <65, and >65 years), and sex of the gambler/gamer.

### Data Analysis

A content text analysis was performed on the corpus using the open-source automated analysis software Iramuteq (version 0.7 alpha 2) according to the Reinart method [26], which is a descending hierarchical classification method to obtain stable classes of words. Classes of words were the most significant themes of the corpus, after a first lemmatization step, when all words (ie, nouns, verbs, adjectives, adverbs) were reduced to their radicals into “forms.” The Reinart method was performed as follows: (i) text segmentation into text segments of approximatively 3 lines; (ii) definition of a contingency table of forms (as defined above) and text segments; and (iii) descending hierarchical classification with a double classification of text segments grouping in an iterative partition that maximizes interclass inertia and minimizes intraclass inertia, so that classes were as homogeneous as possible within the class (ie, having text segments with the common pattern of forms) and as heterogeneous as possible between the classes (having less in common). This iterative partition stopped when the extracted interclass inertia was not improved by a new partition of the corpus [27]. The final number of classes was a priori undetermined. A dendrogram was generated.

Once these classes were identified, associations between classes and “passive” independent variables were tested. Passive independent variables were sociodemographic variables as described above, including gaming and gambling. The strength of association between the forms and the classes, and between the passive independent variables and the classes was determined by *χ^2^* tests. Significant forms and variables are presented (*P*<.01). Given the size of the corpus, we chose to present only forms and variables with *χ^2^*>100 within a class. This automated text-mining approach generates similar themes to traditional qualitative content analysis, and is thus considered to be a reliable text analysis [28]. In this study, we purposely used a computerized automated analysis to limit any a priori assumptions on the burden linked to gambling/gaming and to ignore gambling and gaming categorizations so as to explore commonalities and specificities of these two fields: gaming and gambling variables were the only passive variables as explained above. Classes were then described according to context.

### Ethics

Data collection was anonymous and declared to the National Commission for Data Processing and Liberties (CNIL number 1433300). Contactors were informed of data collection and analysis in the Terms and Conditions section of the website.

## Results

### Sample

**Figure 1:**
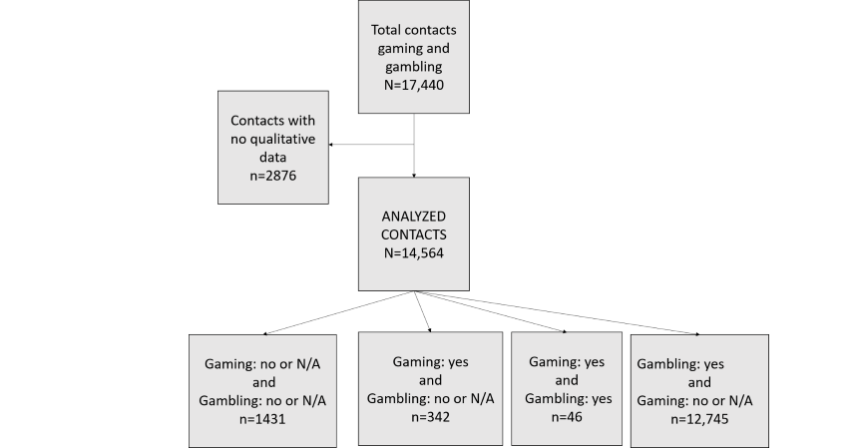
Flow chart of contacts. N/A: not available.

A total of 17,440 contacts were made during the study period, 14,564 (83.51%) of which had written summaries. Figure 1 presents the flow chart of inclusion of contacts in the study. Contactors were predominantly users (10,017/14,564, 68.78%), with 4547 relatives, including 1654 life partners, 1206 parents, 478 children, 398 siblings, 280 friends, 221 other members of the family (179 boy/girlfriends, 35 grandparents, 90 others); 6 were not completed. Users were 58.9% male (n=57 not completed). In total, 1144 were aged <25 years, 7363 were aged 25-64 years, and 703 were aged >65 years (n=5352 not completed). Only 330 contactors (2.27%) contacted the service via internet. Gaming was noted in 388 contacts and gambling in 12,791 contacts.

The mean age of gambling users was 40.9 (SD 17.1) years and that of gaming users was 22.8 (SD 9.1) years. Relatives made up 75.3% of contacts regarding gaming (n=292) and 29.60% regarding gambling (n=3786).

### Statistics of the Corpus

We counted 969,626 occurrences (ie, total number of words) in the corpus, and 19,119 forms and 18,428 analyzed forms. In total, 379 forms showed a frequency ≥100. The top 10 forms (play, game/gamble, to go to, to ask for, money, euro, call, casino, to speak, to wish) were the triggers for making the contact, and gambling was highly represented. The following forms were related to emotional distress: addiction, help-seeking and referral, family members and contexts, financial difficulties, work, and gambling/gaming practice in itself.

### Reinart Descendant Hierarchical Analysis

#### Overview

**Figure 2:**
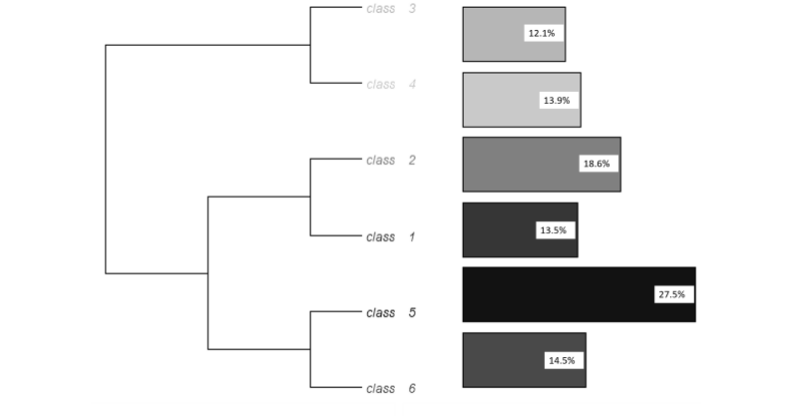
Dendrogram of the Reinart descendant hierarchical analysis.

The corpus was automatically divided into 68,658 text segments. The analysis retained six different classes; 72.7% of segments were classed into one of the six classes. Figure 2 represents the dendrogram of the Reinart descendant hierarchical analysis.

#### Class 1: Gaming Specificities

This class of forms was mostly elicited by relatives of gamers (ie, *χ^2^* values for relatives, gaming, and no gambling were respectively 2629.1, 3869.0, and 1424.6; *P*<.001). The gaming specificities class is characterized by child adverse events such as the death (*χ^2^* for “death”=211.9, *χ^2^* for “dead”=176.6; *P*<.001) or suicide (*χ^2^*=167.9, *P*<.001) of a loved one and the alcohol (*χ^2^*=188.7, *P*<.001) dependence of a parent or divorce (*χ^2^*=101.5, *P*<.001): “addicted to gaming for 8 years, which corresponds to his parents’ divorce” (parent of a male adult gamer via phone contact). Concerns of relatives were driven by missing school (*χ^2^*=184.9, *P*<.001) or a drop in grades (eg, “he’s been missing school more and more, his grades are dropping”; parent of a male gamer via phone contact) and violence (*χ^2^* for violence=219.5, *χ^2^* for violent=479.3; *P*<.001). Violence was significantly associated to this class. Relatives of gamers reported verbal and physical assault on furniture by the gamer, and both hetero- and self-aggressivity:

He had a crisis and became violent toward her [...] he feels she’s depriving him of his freedom. He threatened to hang himself or shoot himself in the head.

Parent of male gamer via phone contact

Daily violence toward her mother as soon as she tries to set limits.

Grandparent of a male gamer via phone contact

They also reported a global violent climate and violence from the parents of the gamer: “she stands between father and son fearing violence against each other” (parent of a male gamer via phone contact).

Although gambling was not associated to this class, some relatives of gamblers also reported violence: “her partner becomes violent and pressures her for money” (life partner of a male gambler via phone contact). Gamblers also reported suicidal thoughts but rather as the consequence of psychological exhaustion:

I swear if someone doesn’t help me I'll hang myself.

Male gambler via internet contact; question

Do you really think that after 11 years of nightmares, I can stop playing? I can’t take it anymore. We are really alone in this battle that we will never win. Obviously, I’ve wanted to kill myself several times.

Male gambler via internet contact; question

#### Class 2: Shared Psychological Distress and Negative Emotions

This class was not significantly associated to gambling or gaming but was associated with relatives, and especially life partners (*χ^2^*=24.9 and 327.5, respectively; *P*<.001), who described both their own distress and the distress of the user (see Textbox 1 for representative quotes).

**Quotes related to psychological distress and negative emotions (class 2).**

**Distress of the user expressed by relatives/life partners:**

“He feels [about his life partner who gambles] a gap, an incomprehension, a feeling of powerlessness” (life partner of a male gamer via phone contact).

“His parents can’t help but support him [financially], as they think that he feels lonely” (sister of a gambler via phone contact).

**Feelings of powerlessness of the user expressed by relatives/life partners:**

“Her husband gambles. When she tries to talk about it, he gets into a terrible rage” (life partner of a male gambler via phone contact).

“She is very angry with her sister-in-law because she gave money to help her and she doesn’t seem to care” (sibling of a female gambler via phone contact).

“The father wants to hit him and restrains himself [since he stole money for video games], the mother is angry and feels hatred toward him, they don’t understand what’s happening” (parent of a male gamer via phone contact).

**Negative emotion and psychological distress expressed by users:**

“He feels empty and feels like he’s lost years just gaming and hasn’t grown up” (male user, unspecified, via phone contact).

“This young woman is very concerned about normality and shows a strong feeling of guilt and shame” (female gambler via phone contact).

“She’s aware that if she thinks she is [addicted to video games], there’s suffering behind it. She can’t figure out why she’s feeling bad” (female gamer via phone contact).

“He feels difficulty in relating to others, inhibition, shame, and guilt about not being able to take part in a discussion. He played video games for 4 years, cutting himself off from others, now feels out of step, with concentration difficulties [...], depressed” (male gamer via phone contact).

“She feels lonely and isolated, the gambling, for the last year, fills her loneliness” (female gambler via phone contact).

Powerlessness is often linked with a lack of trust (*χ^2^*=84.8, *P*<.001): “His life partner has no trust in him” (life partner of a male gambler via phone contact). Anger was also present in this class (*χ^2^*=94.1, *P*<.001), manifested both by users and relatives, and characterizes communication and the relationship between users and their relatives as the outcome of lack of trust and powerlessness (see Textbox 1 for representative quotes).

However, a large number of text segments from this class was derived from contacts with gamblers and gamers themselves. The word “feeling” and several negative emotions such as shame, guilt, and loneliness were associated to this class. Textbox 1 includes quotes representing the panel of negative emotion and psychological distress felt by users.

#### Class 3: Procedure for Being Banned From Gambling

This class was elicited by gamblers (overall *χ^2^*=475.0, to ban *χ^2^*=4199.0, *P*<.001; users, gambling, and no gaming *χ^2^*=325.7, 145.8, and 286.3, respectively; *P*<.001): “He has questions about gambling ban” (male gambler via phone contact).

#### Class 4: Provided Help

The class related to provided help was mostly elicited in contacts with users themselves (overall: *χ^2^*=189.7, *P*<.001; referral, help, address *χ^2^*=2991.4, 973.4, and 1600.3, respectively; *P*<.001): “I support and refer this user toward the clinical outpatient center […] because he says he bets more and more on horse races with less and less control” (male gambler via internet contact; question).

#### Class 5: Gambling Specificities

This class was mostly elicited by gamblers (ie, users and gambling: yes, *χ^2^*=2669.8 and 338.4, respectively; *P*<.001). The words “adrenalin,” “[not being able to] stop,” “can’t,” “control,” “desire to make money,” and “chasing” were significantly associated to this class and reflect symptoms of gambling disorder, especially the core symptom of addiction that is loss of control, and gambling expectations focused on excitement and making money. Textbox 2 includes quotes that illustrate this association.

**Quotes related to gambling specificities expressed by users (class 5).**

“Says she's addicted to gambling, can’t avoid gambling when she walks past a tobacconist’s shop” (female gambler via phone contact).

“He can’t get over the money he’s lost and keeps chasing losses” (male gambler via phone contact).

“He realizes that he is losing control. He is looking for the desire to make money but also the excitement of the risk of losing” (male gambler via phone contact).

“He explains very well what gambling provides: adrenalin, feeling of being surrounded” (male gambler via phone contact).

“Keeps chasing losses, and that’s all he thinks about all the time. He’s fed up and wants to stop” (male gambler via internet contact; chat).

“I'm exhausted, I've spent all my savings on gambling and I can’t stop it's stronger than me... please help me...” (male gambler via internet contact; question).

The words “win” and “lose” were also significantly associated to this class, along with words related to the gambling practice in itself (eg, horse betting, sport betting, poker, scratch card, soccer, money, and to bet).

#### Class 6: Financial Problems

This class was more strongly elicited by relatives than by users themselves (*χ^2^*=887.3, *P*<.001) and barely in gambling (*χ^2^*=28.2, *P*<.001 for gambling and *χ^2^*=13.0, *P*=.003 for no gaming), especially life partners (*χ^2^*=820.0, *P*<.001): “The women is calling about her brother, who she just discovered has a casino addiction and a huge debt. The family is in shock” (sibling of a male gambler via phone contact). Critical financial situations and debts due to gambling were reported repeatedly along with suicidal thoughts: “says he has so many debts [due to gambling] that he feels there is no other solution than suicide” (male gambler via internet contact; chat).

Gamer spending remains anecdotal and repeatedly financed by theft, but does not fall into the category of financial damage: “this young man says that he is addicted to video games, he spends a lot of money and has stolen his grandfather’s credit card” (male gamer via phone contact).

Multimedia Appendix 1 presents the *χ^2^* values (>100) between forms and the classes, and between the passive independent variables and the classes (all *P*<.001).

## Discussion

Our quantitative-qualitative explorative study allowed for exploration without any a priori burdens linked to gaming and gambling in a large and exhaustive sample of contacts to a help service by phone or online. As hypothesized, some classes grouped both gaming and gambling, but others illustrated their specificities, both for the profiles of users and for core clinical symptoms.

Negative emotions described in class 2 regarded gaming and gambling for users and relatives. This class highlights that users or relatives of gamers and gamblers can experience psychological distress related to these behaviors. Current American Psychiatric Association and World Health Organization classifications mention as a prerequisite for gambling and gaming disorder that these behaviors lead “to clinically significant impairment or distress” [1,29], and align with expert consensus that these behaviors produce psychological distress in some people [30]. Our findings support this scientific consensus and the relevance of structuring care and facilitating access to care for these populations, while countering the assumptions of moral panic on gambling and gaming particularly. These negative emotions (especially guilt and loneliness) have been consistently described in addictions, particularly with regard to gambling disorder [31]. The feeling of incomprehension of the user’s own behavior and mental state, and of the user’s behavior regarding relatives had also been previously described in the context of gambling [31]. Our study illustrates that this feeling is shared in gamers and relatives, and is similar to the powerlessness to change or help. Interestingly, “loss of control,” as an escalation of behavior and failure of inhibition, was present in another class specific to gamblers, but was not associated to gaming. Thus, the specificities of gambling go beyond the practical aspect of games as a consumer product, touching on the addictive symptomatology. Loss of control, the core symptom of addiction, was not found to be associated with gaming in this study. This finding questions the recent assertion that gaming disorder is part of the addictive disorder framework in the ICD-11. Although loss of control had been previously described as part of the disease in gaming disorder in qualitative research with a very small sample of nine gamblers using the grounded theory approach [32], our computerized analysis on a very large sample allowed us to move away from a priori models of addiction for understanding gaming and gambling disorders.

The difference in the onset of suicidal thoughts also puts into question the commonality between gaming and gambling disorder. In gambling, suicidal thoughts seemed to occur concomitantly to psychological exhaustion and the feeling of being pushed to the wall due to financial damage. These feelings have been consistently described in the literature [23,33]. In gaming, the suicide theme appeared indirectly during conflicts with parents. This finding supports previous studies suggesting predominant roles in gaming disorder development and maintenance of parental rejection, poor attachment modalities, and resulting poor self-esteem [34]. Moreover, childhood adverse events and a difficult family climate were broadly reported in class 1, which reflects gaming specificities, namely in younger users. Emotional trauma was recently described as an indirect path to gaming disorder through depressive symptoms [35]. The hypothesis of high involvement in gaming as an effort to cope has also been previously debated [36]. No causal inference can be drawn from this cross-sectional study; however, relatives repeatedly described a chronological link between an adverse event and the onset of gaming disorder. This could also reflect a reporting bias in that relatives, and particularly parents who comprised the majority of the gaming specificities class, are more prone to share family history, or that the helpline operatives asked more often about the familial context when the user was a gamer. Communication difficulties with parents were also widely reported. Parents’ anxiety and own depression, previously reported to be highly correlated to gaming disorder in children [37], could contribute to these particularities and especially to communication difficulties.

Relatives of gambling users frequently complained about financial difficulties, grouped in class 6, that often become their shared burden. This result supports previous findings that financial difficulties and familial conflicts were key help-seeking precipitators among gamblers [38]. In contrast, specific help-seeking precipitators in gamers were externalized behavior problems and drop in grades, reported in class 1. Externalized behavior problems have been previously linked to cumulative childhood trauma [39]. A future exploration of this link in gaming disorder could reinforce therapeutic strategies and highlight prevention.

Our study has several limitations. The imbalance between gaming and gambling and the proportion of relatives in the respective subsamples could have influenced the hierarchical classification; however, both gaming and gambling samples were very large compared with those reported in classical qualitative studies. Some contactors could have contacted the help service several times, although the anonymity of the facility prevented documentation of repeat contacts. Moreover, no structured diagnostic or clinical assessments were collected during the calls, and we cannot assume that all contacts regarded people with a clinical addictive disorder. We can only assume that the contactors felt the need to seek help regarding gambling or gaming.

In conclusion, negative emotions and shared distress linked to gambling and gaming support their classification as mental disorders in se, but meaningful differences were observed in core symptoms of addiction between gamers and gamblers, beyond specificities related to the behavior itself. Our findings support differences in the development and maintenance of gaming and gambling disorder and should been further explored in a process-oriented approach.

## Appendix

Multimedia Appendix 1 χ^2^ values (>100, all <italic>P</italic><.001) between forms and the classes, and between passive independent variables and the classes.

## Abbreviations

ICD: International Classification of Diseases
