# Supplementary material for: Burden and Help-Seeking Behaviors Linked to Problem Gambling and Gaming: Observational Quantitative and Qualitative Analysis
Source: JMIR Ment Health. 2021 Nov 26;8(11):e26521. doi: 10.2196/26521 (PMC8665394; doi:10.2196/26521)
Supplement: Multimedia Appendix 1 [file mental_v8i11e26521_app1.docx]

**Multimedia Appendix 1.** *Χ^2^* values (>100, all *P*<.001) between forms and the classes, and between passive independent variables and the classes.

| Form/variable | | *Χ^2^* |
| --- | --- | --- |
| **Class 1: Gaming specificities** | | |
|  | Relative^a^ | 3869.0 |
|  | Relative_parent^a^ | 3021.0 |
|  | Gaming_yes^a^ | 2629.1 |
|  | Sex_female^a^ | 2018.2 |
|  | Father | 1746.1 |
|  | Mother | 1695.2 |
|  | Gambling_no^a^ | 1424.6 |
|  | Son | 1256.5 |
|  | Sister | 768.4 |
|  | Relative_child^a^ | 692.4 |
|  | His | 568.9 |
|  | Video | 551.4 |
|  | Daughter | 543.1 |
|  | Parent | 488.9 |
|  | Violent | 479.3 |
|  | Big | 418.2 |
|  | Relative_siblings^a^ | 416.3 |
|  | Brother | 412.7 |
|  | Child | 397.6 |
|  | Relative_family others^a^ | 391.5 |
|  | Live | 341.5 |
|  | Worried | 340.5 |
|  | She | 301.2 |
|  | Beautiful | 282.0 |
|  | Brothers | 257.5 |
|  | Caller | 231.9 |
|  | Violence | 219.5 |
|  | Death | 211.9 |
|  | Relative grand_parent^a^ | 206.0 |
|  | Student | 199.6 |
|  | Year | 190.2 |
|  | Alcoholic | 188.7 |
|  | School | 184.9 |
|  | Studies | 181.1 |
|  | Death | 176.6 |
|  | Room | 171.8 |
|  | Suicide | 167.9 |
|  | Was | 166.6 |
|  | Network | 159.9 |
|  | With | 159.0 |
|  | Mum | 156.4 |
|  | Computer | 154.2 |
|  | His | 143.5 |
|  | Boy | 138.3 |
|  | Childhood | 132.9 |
|  | At | 130.7 |
|  | Family | 130.2 |
|  | Their | 129.0 |
|  | His | 124.4 |
|  | School | 120.2 |
|  | Take care of | 118.4 |
|  | Media_phone^a^ | 110.6 |
|  | Cancer | 110.5 |
|  | Dead | 109.7 |
|  | Elder | 108.7 |
|  | House | 101.6 |
|  | Divorce | 101.5 |
|  | Refuse | 101.3 |
|  | Home | 100.5 |
| **Class 2: shared psychological distress** | |  |
|  | Feel | 359.8 |
|  | Thing | 333.6 |
|  | Relative_life partner^a^ | 327.5 |
|  | Speak | 284.1 |
|  | Life | 259.7 |
|  | See | 232.5 |
|  | Gaming_na^a^ | 223.7 |
|  | Gambling_na^a^ | 216.7 |
|  | Solitude | 207.1 |
|  | Not | 205.0 |
|  | This is | 202.2 |
|  | Need | 179.7 |
|  | Someone | 159.8 |
|  | Feel | 159.2 |
|  | Difficult | 158.9 |
|  | Feeling | 155.9 |
|  | Than | 155.7 |
|  | Not | 153.3 |
|  | Seem | 153.0 |
|  | Desire | 152.4 |
|  | Situation | 152.0 |
|  | Anguish | 151.3 |
|  | Say | 149.2 |
|  | Relationship | 146.7 |
|  | Even | 136.9 |
|  | Shame | 136.2 |
|  | Feeling | 135.2 |
|  | Change | 134.0 |
|  | Understand | 133.0 |
|  | Put | 127.6 |
|  | Alone | 125.3 |
|  | Think | 114.7 |
|  | Hear | 112.2 |
|  | Miss | 111.6 |
|  | Approach | 110.8 |
|  | Go to | 108.8 |
|  | Than | 108.8 |
|  | Suffering | 108.7 |
|  | Try | 106.5 |
|  | Very | 105.9 |
|  | Sex_female^a^ | 100.2 |
| **Class 3: ban from gambling procedure** | |  |
|  | Ban | 4745.0 |
|  | Ban | 4199.0 |
|  | Casino | 2528.0 |
|  | Website | 1910.7 |
|  | Procedure | 1619.4 |
|  | Ask for | 1595.1 |
|  | Ministry | 1040.3 |
|  | Do | 1037.6 |
|  | Age_na^a^ | 958.6 |
|  | Get to know | 925.7 |
|  | Play | 919.2 |
|  | Volunteer | 869.1 |
|  | Regulation authority | 811.3 |
|  | Interior | 584.3 |
|  | Access | 560.9 |
|  | Online | 498.5 |
|  | Answer | 491.8 |
|  | Remove [a ban] | 488.4 |
|  | Wish | 484.8 |
|  | Mail | 461.0 |
|  | On | 440.0 |
|  | Step | 436.8 |
|  | Ones' | 425.2 |
|  | Mail | 402.4 |
|  | Send | 379.7 |
|  | Modalities | 338.7 |
|  | Relative_na^a^ | 326.7 |
|  | User_type^a^ | 325.7 |
|  | Send | 324.5 |
|  | Inform | 301.0 |
|  | From | 299.9 |
|  | French lottery | 290.4 |
|  | Ban | 286.7 |
|  | Gaming_no^a^ | 286.3 |
|  | Contact | 276.6 |
|  | Letter | 276.3 |
|  | Info | 262.3 |
|  | Steps | 253.6 |
|  | Correspondent | 243.7 |
|  | Number | 240.3 |
|  | Register | 238.7 |
|  | Want | 230.0 |
|  | Winamax | 209.4 |
|  | Measure | 201.8 |
|  | Auto | 200.5 |
|  | Information | 199.2 |
|  | Identity | 190.1 |
|  | Ban | 182.2 |
|  | French | 177.8 |
|  | Betclic | 168.9 |
|  | How | 168.4 |
|  | Internet | 147.9 |
|  | Gambling_yes^a^ | 145.8 |
|  | Banned | 144.2 |
|  | Exclude | 137.8 |
|  | Police station | 133.2 |
|  | Address | 128.8 |
|  | Written | 128.7 |
|  | For | 122.2 |
|  | Carry out | 117.7 |
|  | Walk | 115.7 |
|  | Fraud | 113.9 |
|  | Police | 106.0 |
| **Class 4: help provided** | |  |
|  | Wish | 2382.3 |
|  | Direct | 2211.4 |
|  | Address | 1600.3 |
|  | Out-patient department (csapa) | 1558.3 |
|  | Orientation | 1460.8 |
|  | To | 1222.9 |
|  | Help | 973.4 |
|  | Center | 870.0 |
|  | Call | 741.3 |
|  | Dependence | 694.1 |
|  | Care | 598.0 |
|  | Service | 561.4 |
|  | Call back | 558.2 |
|  | Stop | 551.2 |
|  | Organization | 543.9 |
|  | Info | 539.1 |
|  | For | 526.3 |
|  | Inform | 472.1 |
|  | Consultation | 455.3 |
|  | Help | 444.7 |
|  | Play | 443.4 |
|  | Man | 385.7 |
|  | Load | 385.7 |
|  | Gaming_no^a^ | 370.6 |
|  | Player | 362.2 |
|  | Be | 359.0 |
|  | Device | 333.3 |
|  | Call | 306.2 |
|  | Support | 302.7 |
|  | Treated | 270.7 |
|  | Age_na^a^ | 263.9 |
|  | Addiction | 251.7 |
|  | Specialised | 249.4 |
|  | I | 244.7 |
|  | Listen to | 239.1 |
|  | Mr. | 236.2 |
|  | Search | 233.7 |
|  | Support | 229.2 |
|  | Give | 226.7 |
|  | Place | 224.3 |
|  | Reminder | 221.4 |
|  | Group | 217.4 |
|  | Our | 204.3 |
|  | Exist | 196.2 |
|  | Speech | 195.8 |
|  | Relative_na^a^ | 190.6 |
|  | User^a^ | 189.7 |
|  | Need | 183.0 |
|  | Demand | 180.8 |
|  | Number | 169.6 |
|  | Propose | 166.6 |
|  | Contact | 165.6 |
|  | Encourage | 159.0 |
|  | Information | 158.3 |
|  | Who | 154.4 |
|  | Advice | 153.9 |
|  | Addict | 150.9 |
|  | Act | 150.0 |
|  | Call | 135.1 |
|  | Discuss | 134.3 |
|  | Mention | 132.1 |
|  | Consult | 131.4 |
|  | Support | 129.7 |
|  | Speak | 128.4 |
|  | Hang up | 128.0 |
|  | Wish | 127.7 |
|  | Psy | 127.5 |
|  | Research | 127.0 |
|  | Meet | 127.0 |
|  | Woman | 124.3 |
|  | Report | 123.4 |
|  | Already | 120.6 |
|  | Ask for | 119.6 |
|  | On | 116.0 |
|  | Regarding | 114.8 |
|  | Addiction | 114.1 |
|  | Sex_male^a^ | 113.9 |
|  | Indicate | 106.6 |
|  | Possibility | 105.0 |
|  | Technique | 103.1 |
|  | Appointment | 103.0 |
|  | Take | 101.3 |
|  | Psychological | 101.0 |
|  | Invite | 100.7 |
| **Class 5: gambling specificities** | |  |
|  | Relative_na^a^ | 2678.9 |
|  | User^a^ | 2669.8 |
|  | Win | 2359.5 |
|  | Play | 1805.9 |
|  | Sex_male^a^ | 1765.0 |
|  | Lose | 1060.1 |
|  | Euro | 870.1 |
|  | Age_young^a^ | 700.5 |
|  | Bet | 688.8 |
|  | Sport [betting] | 654.0 |
|  | He | 565.4 |
|  | Day | 430.0 |
|  | Money | 413.6 |
|  | Win | 413.3 |
|  | Big | 392.3 |
|  | Start | 343.0 |
|  | Gambling_yes^a^ | 338.3 |
|  | Scratching | 329.9 |
|  | From | 292.5 |
|  | Amount | 292.3 |
|  | Football | 277.5 |
|  | Week | 243.9 |
|  | Lottery | 243.3 |
|  | Times | 230.7 |
|  | Bar | 213.5 |
|  | Bet | 202.7 |
|  | Month | 188.6 |
|  | Can/can’t | 187.8 |
|  | Tobacco | 186.8 |
|  | Match | 180.7 |
|  | Horse betting | 167.6 |
|  | Live lottery | 161.6 |
|  | Stop | 151.9 |
|  | Spend | 150.2 |
|  | Horse | 150.0 |
|  | Replay | 135.3 |
|  | Put | 134.2 |
|  | Desire to make money | 128.2 |
|  | Buy | 125.3 |
|  | Chase | 123.5 |
|  | Age_adult^a^ | 122.5 |
|  | Work | 118.8 |
|  | Pleasure | 115.9 |
|  | Stop | 114.2 |
|  | Hope | 112.3 |
|  | Year | 107.1 |
|  | Adrenaline | 97.7 |
| **Class 6: financial problems** | |  |
|  | Debt | 1252.1 |
|  | Credit card | 1107.3 |
|  | Reimburse | 1040.1 |
|  | Pay | 1033.8 |
|  | Euro | 935.4 |
|  | Bank | 889.9 |
|  | Relative^a^ | 887.3 |
|  | Loans | 871.0 |
|  | Account | 852.5 |
|  | Relative_life partner^a^ | 820.0 |
|  | Loan | 794.8 |
|  | Rent | 759.0 |
|  | Bank | 618.0 |
|  | Visa card | 587.0 |
|  | Discovered | 568.4 |
|  | Contract | 370.7 |
|  | Month | 339.6 |
|  | Loan | 271.7 |
|  | Sex_female^a^ | 270.2 |
|  | Invoice | 268.1 |
|  | Steal | 263.7 |
|  | Income | 259.8 |
|  | Spend | 235.7 |
|  | Realize | 214.2 |
|  | Husband | 166.4 |
|  | Use | 162.8 |
|  | Bank statements | 160.6 |
|  | Cheques | 157.4 |
|  | Have | 148.7 |
|  | His | 143.5 |
|  | Debt | 138.3 |
|  | Taxes | 133.1 |
|  | Late | 127.1 |
|  | Name | 118.2 |
|  | Debt | 117.6 |
|  | Money | 116.7 |
|  | Come | 109.3 |
|  | On loan | 108.9 |
|  | Loan | 105.6 |
|  | Organization | 105.6 |

^A^passive independent variable.
